# Indirect genomic effects on survival from gene expression data

**DOI:** 10.1186/gb-2008-9-3-r58

**Published:** 2008-03-22

**Authors:** Egil Ferkingstad, Arnoldo Frigessi, Heidi Lyng

**Affiliations:** 1 Department of Biostatistics and (sfi) Statistics for Innovation, University of Oslo, Gaustadalleen, Oslo, NO-0314, Norway; 2 Centre for Integrative Genetics, Norwegian University of Life Sciences, Arboretveien, Aas, NO-1432, Norway; 3 Department of Radiation Biology, Institute for Cancer Research, Norwegian Radium Hospital, Montebello, Oslo, NO-0310, Norway

## Abstract

In cancer, genes may have indirect effects on patient survival, mediated through interactions with other genes. Methods to study the indirect effects that contribute significantly to survival are not available. We propose a novel methodology to detect and quantify indirect effects from gene expression data. We discover indirect effects through several target genes of transcription factors in cancer microarray data, pointing to genetic interactions that play a significant role in tumor progression.

## Abstract 2

A novel methodology is presented for detecting and quantifying indirect effects on cancer survival mediated through several target genes of transcription factors in cancer microarray data.

## Background

There exists a large literature studying associations between survival and high throughput gene expression data [1-5]. Also, much work has been done to elaborate pathways and regulatory networks [6-10]. We have developed a new method combining survival and pathway analysis technologies, aiming at a causal understanding of how gene expression affects survival. This allows us to discover indirect effects of gene expression on patient survival, mediated through other genes. To our knowledge, no comparable method exists that can achieve this. For the first time, we are able to identify in cancer microarray data significant indirect effects of transcription factors, such as PPAR proteins, *E2F1 *and *MYC*, on survival.

Genome-wide exploration for genes involved in malignant diseases will enable the development of new approaches in cancer diagnostics and therapeutics that will revolutionize the drug discovery field and the development of personalized medicine [11,12]. Lists of genes predictive for treatment outcome of various cancers have been presented, and may potentially be used for selecting patients at risk for treatment failure and aid in clinical decision making. However, the organization of the prognostic genes into structured, functionally meaningful information is difficult and, currently, one of the main obstacles limiting the clinical utilization of microarray data [13,14].

A major challenge in the interpretation of microarray results is understanding the biological effect mediated by transcription factors. These proteins are often key actors in complex regulatory networks containing many signaling pathways, and may interact with other prognostic genes. They can have several modes of interaction with their targets, such as transcriptional activation and/or repression of genes and post-transcriptional modification of proteins [15,16]. Their effect can, therefore, be mediated both by changing the expression level of other genes and through mechanisms undetectable in gene expression studies. Due to the central role of many transcription factors in controlling the cellular phenotype, these have been proposed as potential targets for therapeutic intervention [17]. However, transcriptional interaction between these proteins and other genes makes it difficult to predict the outcome of such interventions. Elucidation of how the different effects mediated by transcription factors contribute to the development of aggressive cancer phenotypes will aid the design of efficient drugs that interfere with key pathways of the regulatory network.

Current pathway analysis tools have proved useful for validating known interactions of transcription factors and proposing unknown pathways in their regulatory networks [18]. However, these tools make no use of the important information represented by patient survival data and are not, therefore, suitable for exploring direct transcription factor-target relationships that may have prognostic value. Our aim was to enable detection, separation, quantification and comparison of possible direct and indirect effects on survival that are mediated by transcription factors. We consider a data set with genes, the expression levels of which are measured using material from patients. Note that the data consists of both the gene expression measurements and a data set of regulatory interactions between genes. A gene has an 'indirect effect' on survival if its expression influences survival through one or more other prognostic genes present in the data. A gene has a 'direct effect' on survival if its expression influences survival and no other gene is found in the dataset through which this effect is mediated. A direct effect is caused by interactions that are undetectable in the given gene expression data, because the effect is mediated either through protein modifications or by transactivation/repression of genes that are not associated with survival and/or are not included in the data set.

We applied the method to the gene expression data of three previously published cancer studies. In all three cases we identified several transcription factors with one or more indirect effects on survival, pointing to the interactions of major importance for the development of an aggressive tumor phenotype. Although the indirect effects were always weaker than the direct effect, they are highly significant and of biological interest. We further demonstrate that the indirect effect did not always strengthen the direct effect, but for some genes, counteracted it, posing fundamental questions about the effect of therapeutic targeting of transcription factors. Protein expression, phosphorylation and/or enzymatic activities can be used alone or together with gene expression in our model, providing a more comprehensive exploration of the pathways. Our method represents a totally new way of utilizing large scale gene and protein data that may increase our knowledge of how specific transcription factors contribute to the progression and treatment outcome of cancers as well as other diseases.

## Results

### Hunting for indirect effects

**Figure 1:**
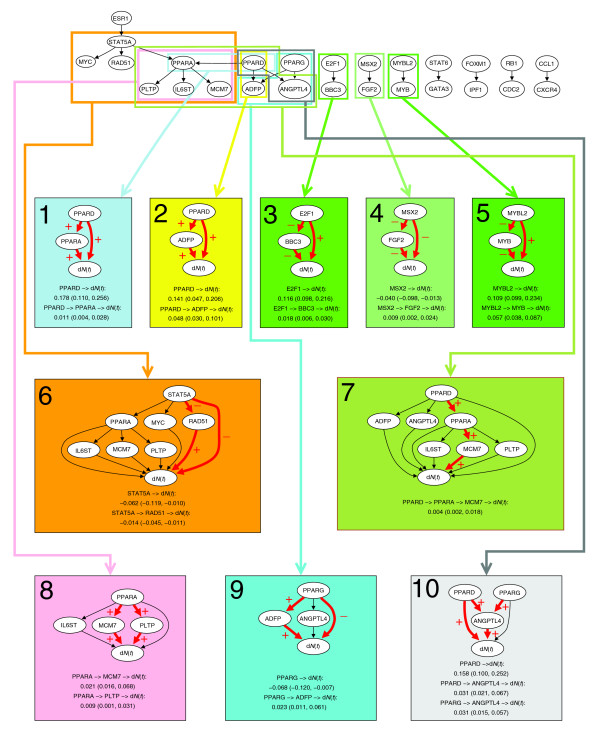
Dynamic path models for the Dutch breast cancer data set. The top panel shows the thinned survival forest after selecting genetic interactions for which an indirect and direct effect likely existed. Black arrows indicate a total of 19 significant interactions. The thinned forest consisted of eight networks. A number of dynamic path models were fitted to different sub-networks of these networks: Each connected component, each rooted subtree (that is, each gene with all of its descendants), and each interaction separately. For ten models there was at least one significant indirect effect, indicated with rectangles of different colors. Below the thinned survival forest, the ten models with at least one significant indirect effect are shown. Interactions with significant direct or indirect effects are marked with red arrows. The plus and minus signs on arrows between two genes indicate transcriptional activation and repression, respectively, whereas the plus and minus signs on arrows pointing to survival (d*N*(*t*)) indicates that poor survival is associated with activation and repression of the gene, respectively. For each significant path, the average strength of the direct and indirect effect during the first five years is listed, along with a 95% bootstrap confidence interval.

First, we illustrate the results that are obtained with our method, using the genes *PPARD *(encoding peroxisome proliferator-activated receptor D) and *ADFP *(encoding adipose differentiation-related protein) as an example (Figure 1, model 2). All details are explained in the subsequent text. We have gene expression data for both genes from cancer patients and censored survival data from the same patients. It is known that expression of *PPARD *influences expression of *ADFP*. An effect of *PPARD *on survival could, therefore, be mediated through *ADFP*. In our terminology, this is an indirect effect of *PPARD *on survival, through *ADFP*. Other indirect effects of *PPARD*, through other genes, could also exist, and *PPARD *could also have a direct effect on survival, that is, an effect that is not mediated through any other genes in our data set. Using our method, we can discover and quantify the strengths of such indirect and direct effects. Specifically, we found that, summed over the first five years, *PPARD *had a direct effect on survival of 0.141 (with a 95% bootstrap confidence interval of (0.047, 0.206)), and an indirect effect of 0.048 (95% confidence interval of (0.030, 0.101)). In this case, all effects are positive, indicated by plus signs on the arrows in Figure 1. In other cases, the effects can be negative, indicated by minus signs. Positive effects are harmful (increase the risk of death), while negative effects are beneficial. Since the bootstrap confidence intervals do not contain zero, both the direct and indirect effects are significant. The 'total effect' is simply the sum of the direct and indirect effects. Here, approximately 24% of the total effect is indirect.

We developed a stepwise procedure, generating the candidate networks, selecting significant genetic interactions, and identifying the most relevant dynamic path models with indirect effects.

#### Survival genes and survival forests

**Figure 2:**
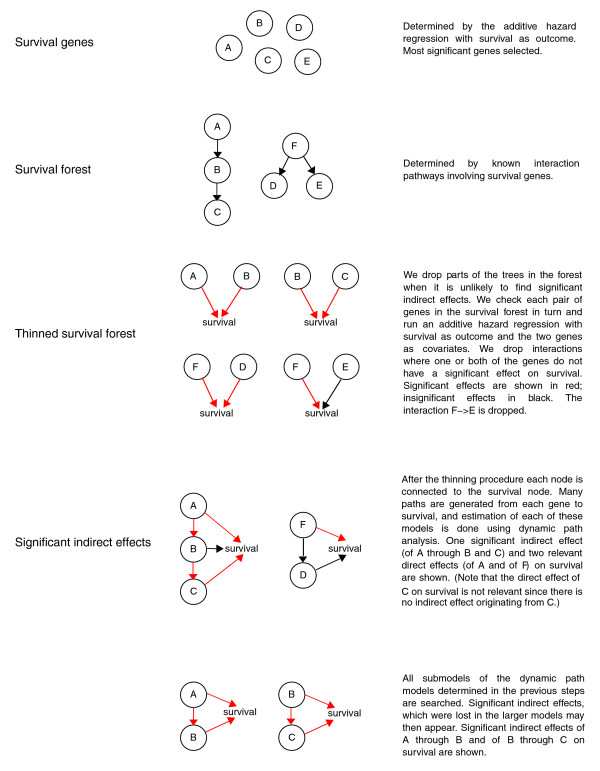
Selecting dynamic path models. This figure shows a description of the dynamic path model selection procedure. A, B, ... represent gene *A*, gene *B*, .... Arrows indicate interactions between genes or between gene and survival.

To compile a first list of genes associated with survival, we used a simple univariate selection procedure: for each gene in a data set, an additive hazard regression model was estimated with the gene expression value as the only covariate (Figure 2). The genes were then ranked according to their statistical significance (*p*-value), and a set of these top genes, called survival genes, was considered further. *P*-values were calculated using the common test for effects in the additive hazard model, as described in [19]. Any rule can be used to select survival genes from the full data set, for example, thresholding according to *p*-value or number of genes. More complex multivariate selection procedures could also be used [5], but in this context we believe that they would not be advantageous. Since the aim was to identify genes highly correlated with survival, we wanted all genes of this type to be retained by the selection procedure, even if they are correlated to each other. Stepwise selection or penalized regression methods model dependence between genes, and hence lead to rankings that do not have this property. Most importantly, the set of survival genes must be large enough to ensure a rich survival forest in the continuing analysis. We then input the survival genes into Pathway Studio [8], which generates pathways involving the survival genes based on public databases and published literature. The use of Pathway Studio is in no way essential to our methodology. The only requirement is that the hypothesized pathways can be modeled by directed graphs. We obtained a collection of directed graphs, called a 'survival forest', representing known pathways involving the survival genes (Figure 2). Only pathways that could be represented as directed acyclic graphs (DAGs) were selected. Our method currently does not handle feedback effects, which are then appropriately simplified. Since our basis was gene expression data, we considered only transcriptional interactions, meaning that each pathway contained at least one protein with known transcription factor activity interacting with one or more other genes by changing their expression level. Each interaction was then of the form gene *A *→ gene *B*, which we write as *A *→ *B*, representing that the expression of gene *A *influences the expression of gene *B*. The collection of all the pathways in the survival forest was analyzed further, to find the significant direct and indirect effects on survival.

#### Thinning the survival forest for possible indirect effects

Since our purpose was to identify transcription factors with one or more indirect effects on survival, in addition to direct effects, we deleted all genes where significant indirect effects were unlikely. This selection was based on the likelihood of finding evidence of indirect effects (Figure 2). For each interaction *A *→ *B *the additive hazard regression model with *A *and *B *as covariates and survival as response was fitted to the gene expression data. We chose the interactions for which both the effects of *A *and *B *on survival were significant at *p *< 0.05 and dropped other links. This was done because the interaction *A *→ *B*, for which both *A *and *B *influence survival, gives the potential for an indirect effect of *A *through *B *in addition to the direct effect of *A *on survival. The selection procedure, therefore, reduced the survival forest to a collection of interaction networks for which the expression of all genes was significantly correlated with survival. Thinning also leads to a computational advantage. This 'thinned survival forest' formed the basis for the dynamic path modeling.

#### Selecting dynamic paths with indirect effects

We now searched every network in the thinned survival forest for significant indirect effects by dynamic path analysis [20] (in Materials and methods). This led to a further reduction of the forest, such that it only included networks where indirect effects were significant (Figure 2).

The analysis was performed on each network separately. The results depended on which genes of each network were included in the model. There is a trade-off between accuracy and power when selecting models. Choosing a large model reduces the risk of leaving out possible interacting survival genes. On the other hand, interesting effects may be reduced in a large model, because covariates can be more correlated by chance. Hence, we operated systematically. First, a dynamic path model was fitted to each connected component of the networks separately. Within a connected component, a model was fitted for each gene together with all its descendants (if any). In the final stage of this strategy, each pair of interactions was modeled separately.

For each model, the strength of the individual interactions was precisely quantified as described in Materials and methods. These estimated effects can be positive or negative. For interactions between genes, a positive effect of *A *→ *B *means that an increase in the expression of *A *leads to an increase in the expression of *B*, and a negative effect means the opposite. For an effect from a gene to survival, a positive effect is harmful (increases the risk of death), while a negative effect is beneficial. The unit of the effect is the increase in the death rate per unit increase of gene expression.

After the models had been fitted, we used bootstrapping to judge whether the estimated effects were significant. A total of 1,000 bootstrap replications were used. Because of deaths and censorings, the set of patients on which the estimation is based changes over time. The effects can, therefore, be estimated at every time point and change when the population at risk changes. Hence, the significance of the effects also changes at each time point. We considered an effect as significant if the 95% bootstrap confidence interval did not contain zero after five years, which is a commonly used horizon in cancer studies. Longer time periods can be used, but estimation becomes less precise due to the lower number of patients with such long survival times. We selected only models containing at least one significant effect.

#### Multiple testing

Running a separate test on each genetic interaction created multiple testing concerns. To address these, we used a permutation approach where the whole selection procedure was run repeatedly on randomly permuted survival data. In this way we could assess how many interactions would be found if the gene expression levels and survival times were completely unrelated. A total of 1,000 permutations were run for each data set, and the resulting number of interactions selected when only generated by chance was compared to the actual findings, as demonstrated in Table 1 for the data sets analyzed below.

**Table 1:** Permutation test

Number of interactions	1	2	3	4	5	6	7	8	9	10	11	Our finding
Dutch data	0.844	0.087	0.036	0.017	0.009	0.003	0.003	0.001	0.000	0.000	0.000	19
Uppsala data	0.698	0.150	0.081	0.032	0.018	0.006	0.006	0.002	0.003	0.002	0.002	7
DLBCL data	0.829	0.095	0.043	0.022	0.004	0.005	0.002	0.000	0.000	0.000	0.000	9

This table shows the probabilities of finding the number of interactions listed in the first line, if survival and gene expression were associated at random.

#### Confounding

**Figure 3:**
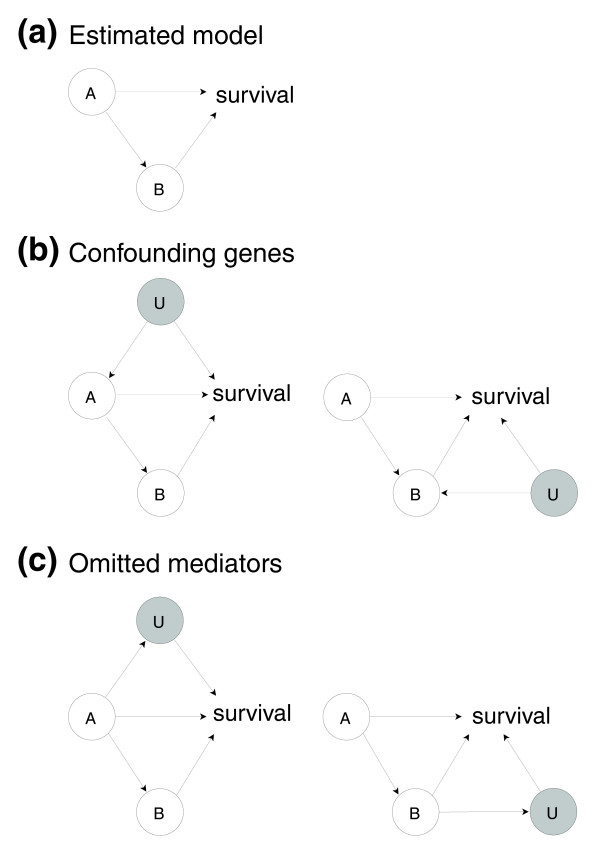
Confounding and omitted mediators. This figure illustrates issues connected to omitted genes/interactions. **(a) **The assumed estimated model, as produced by our method. **(b) **The problem of confounding. **(c) **Two cases of omitted mediators.

Can confounding misguide our results? What if relevant genes or interactions were incorrectly omitted from our models? Figure 3 illustrates this issue. Assume that we would obtain a significant estimated model, with genes *A *and *B *significantly associated with survival, and with the interaction *A *→ *B *present in the thinned survival forest (Figure 3a). A has a direct effect on survival, as well as an indirect effect through *B*. Figure 3b illustrates the problem of confounding. *U *is another gene, or more generally, a collection of genes. The gray shading indicates that *U *is omitted, that is, not a part of the estimated model. The problem is that the 'common cause' *U *will generate, unconditionally on *U*, a statistical association between *A *and survival that is not due to the direct effect of *A *on survival. If the true state of nature corresponds to Figure 3b, while our estimated model is that of Figure 3a, we produce biased effects or a false positive. Assume that the data source of regulatory interactions contains the interactions *U *→ *A *and *U *→ *B*. Then, we argue that the situations in Figure 3b are unlikely to occur in our methodology, because of the way the stepwise selection procedure works (Figure 2). To see this, note that for a confounding gene *U *to be present, *U *must have an effect on survival. But this means that *U *would have been one of the 'survival genes' kept in the first step of the selection procedure, and hence would not be omitted. At least for the breast cancer data sets, we do have expression measurements for the majority of genes that could affect survival. Furthermore, the interaction *U *→ *A *(or *U *→ *B*) would have remained after the thinning procedure, since there would be evidence for both *A *→ survival and *U *→ survival from the data. For these reasons, it appears unlikely that we would estimate the model of Figure 3a if any of the models in Figure 3b were true. In the presence of a confounding gene *U*, the effects *U *→ *A *and *U *→ survival would be discovered, and the correct model would be estimated. However, it should be pointed out that if the interactions *U *→ *A *or *U *→ *B *are not present in the data source one is using (that is, if these regulatory interactions are not known in the literature), then the preceding argument does not hold. Also, if *U *is not a gene, but some unmeasured environmental factor such as smoking, then, as smoking could affect both gene *A *(gene *B*) and survival, the problem of confounding could arise. But this is a potential problem in any statistical analysis not controlling for relevant environmental factors, and nothing in our methodology would make our results more vulnerable than usual to confounding in this more general sense. Still, care should be taken in the interpretation of our models, and we do not claim to discover 'causal relations' in the strict sense of the term. The third general effect of 'missing interactions' is illustrated in Figure 3c. Here, *U *is a (set of) omitted mediator(s). In the left panel, there is an additional path *A *→ *U *→ survival, which is left out of the models, and the left panel shows a case with a missing interaction *A *→ *B *→ *U *→ survival. In fact, this situation is not problematic: in the situation shown in the left panel, the direct effect should be defined as the sum of the two paths *A *→ survival and *A *→ *U *→ survival, and the indirect effects should be defined similarly as the sum of the two paths through *B*. The reason is simply that the inclusion of omitted mediators is equivalent to looking at a system in greater detail (finer resolution), which may always be done, and this does not invalidate the model defined at a coarser resolution.

### Dynamic path model in cancer genomics data

We applied dynamic path analysis on three microarray data sets containing right-censored survival times for the patients. In all cases, we estimated cumulative effects after five years; that is, the effects are sums over the first five years of observation.

#### Dutch breast cancer data

The Dutch breast cancer data set from the study of van de Vijver *et al*. [21] and van Houwelingen *et al*. [22] consists of 24,885 gene expression values for 295 women with breast cancer. A total of 175 genetic pair interactions were generated by Pathway Studio based on the gene list of 1,000 survival genes. Out of these, the selection procedure resulted in 19 interactions for which an indirect and direct effect likely existed (Figure 1). This gave a thinned survival forest with eight networks. The number of 19 interactions is highly significant, showing the pronounced reliability of the results, since in the permutation test a single interaction was selected in 844 out of 1,000 permutations, and more than 8 interactions were never selected (Table 1).

Dynamic path modeling based on the selected genetic interactions of the thinned survival forest resulted in ten models with at least one significant indirect effect on survival (Figure 1). There were two major types of models. The simple models involved two genes in the significant subnetwork, a transcription factor with a single interacting gene (models 1-6 and 9). In the complex models with three or more genes in the significant subnetwork, a transcription factor showed indirect effects through two genes (models 7 and 8), or two transcription factors had an indirect effect through a common gene (model 10). In the former cases the indirect effect was either through serially interacting genes (model 7) or genes interacting in parallel with the transcription factor (model 8). Members of the peroxisome proliferator-activated receptors (PPAR) family were involved in all the complex and some of the simple models, whereas *E2F1*, *MSX2*, and *MYBL2 *were involved in simple models.

In most cases the indirect effect strengthened the direct one, leading to a stronger total effect than suggested from the direct effect. A typical example is shown in model 1, where activation of *PPARD *led to a direct effect of 0.178 and an indirect effect of 0.011 through *PPARA*, resulting in a total effect of 0.189. This means that a unit increase in the expression of *PPARD *implies an increase in the death rate of 0.189 deaths per year, so here the indirect effect is 5.8% of the total effect on survival. The indirect effect could, however, also counteract the direct effect (models 4 and 9). Hence, repression of *PPARG *led to a negative direct effect of -0.068, whereas activation of *PPARG *was indirectly associated with poor survival through *ADPF *with a positive strength of 0.023 (model 9). The total effect of *PPARG *in this model was, therefore, -0.045, still negative but weaker than expected from the direct effect alone. For all models that included both a significant direct and a corresponding significant indirect effect, the indirect effect was weaker than the direct one, but could still represent a strength of more than 50% of the indirect effect (range 6-52%). However, for some models we found indirect effects without corresponding significant direct effects: the indirect effect of *PPARD *in model 7, the indirect effect of *PPARA *in model 8 and the indirect effect of *PPARG *in model 10, suggesting that the indirect effects were strong compared to the direct ones in these cases.

**Figure 4:**
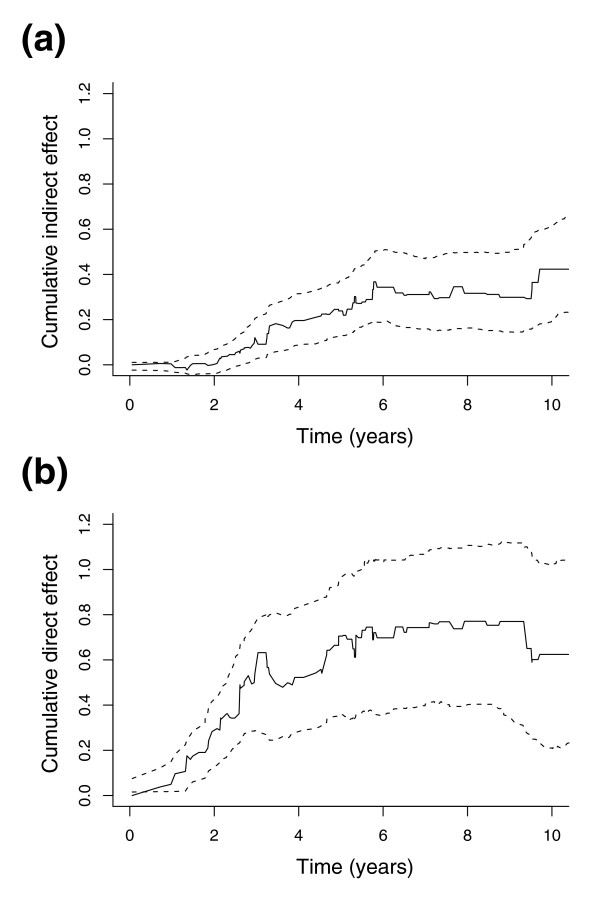
Time evolution of the dynamic path model containing *PPARD *and *ADFP*. This figure shows the time evolution of the model from model 2 of Figure 1. **(a) **The cumulative indirect effect of *PPARD *(through *ADFP*) on survival, and **(b) **the cumulative direct effect of *PPARD *on survival. The indirect and direct effects are estimated as explained in Materials and methods; see particularly equations 3 and 4 for details of the calculations. The indirect effect is approximately constant for the first six years, and zero thereafter (recall that the plots are cumulative). Similarly, the direct effect remains positive and stable for the first three years, and then becomes zero. As expected, confidence intervals become wider over time, due to fewer remaining patients. Based on these plots, the use of a five year horizon seems reasonable.

We have reported estimated cumulative effects after five years. In fact, all our estimates are available in continuous time. To illustrate this, Figure 4 shows the time course development of model 2 of Figure 1, containing the two genes *PPARD *and *ADFP*. From these cumulative plots, we read that the indirect effect (Figure 4a) is positive and stable for the first six years, disappearing thereafter. The direct effect (Figure 4b) is stably positive for the first three years, and then vanishes. Both plots show widening confidence intervals over time, due to fewer remaining patients alive and under observation.

#### Uppsala breast cancer data

**Figure 5:**
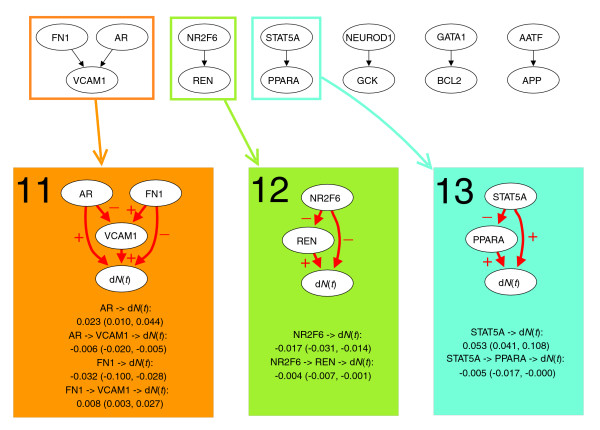
Dynamic path models for the Uppsala breast cancer data set. The top panel shows the thinned survival forest after selecting genetic interactions for which an indirect and direct effect likely existed. Black arrows indicate a total of seven significant interactions. The thinned forest consisted of six networks. A number of dynamic path models were fitted to different sub-networks of these networks: each connected component, each rooted subtree (that is, each gene with all of its descendants), and each interaction separately. For seven models there was at least one significant indirect effect, indicated with rectangles of different colors. Below the thinned survival forest, the seven models with at least one significant indirect effect are shown. Interactions with significant direct or indirect effects are marked with red arrows. The plus and minus signs on arrows between two genes indicate transcriptional activation and repression, respectively, whereas the plus and minus signs on arrows pointing to survival (d*N*(*t*)) indicate that poor survival is associated with activation and repression of the gene, respectively. For each significant path, the average strength of the direct and indirect effect during the first five years is listed, along with a 95% bootstrap confidence interval.

The Uppsala breast cancer data set from Miller *et al*. [23] consists of 44,928 gene expression measurements for 251 breast cancer patients. A total of 380 genetic interactions were generated based on an input list of 2,000 survival genes. Seven interactions in six networks were chosen by the selection procedure (Figure 5). The number of interactions was much higher than expected by chance alone (Table 1), suggesting the selected interactions are highly reliable. The genetic interaction *STAT5A *→ *PPARA *was among those selected, as in the case of the Dutch breast cancer data set (Figure 1).

Three models with at least one indirect effect on survival were found by the dynamic path analysis (Figure 5). All models also included a significant direct effect. There was one complex model, where both *AR *and *FN1 *had an indirect effect through *VCAM1 *(model 11), and two simple models, where *NR2F6 *and *STAT5A *showed indirect effects through *REN *and *PPARA*, respectively (models 12 and 13). The indirect effect of *NR2F6 *strengthened the direct one (model 12), whereas for *AR*, *FN1 *and *STAT5A*, a weakening of the direct effect occurred (models 11 and 13). The strength of the indirect effect ranged from 9-26% of the direct effect.

#### Diffuse large B-cell lymphoma data

**Figure 6:**
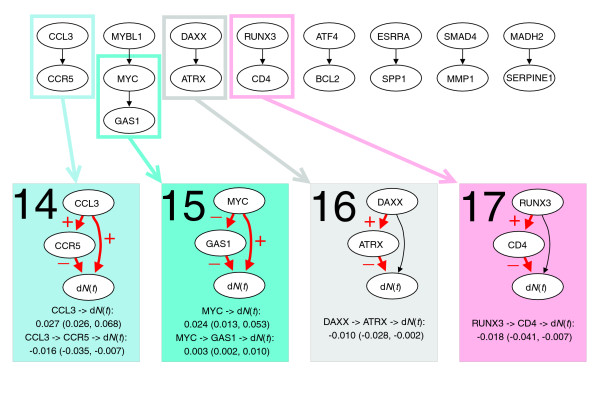
Dynamic path models for the DLBCL data set. The top panel shows the thinned survival forest after selecting genetic interactions for which an indirect and direct effect likely existed. Black arrows indicate a total of nine significant interactions. The thinned forest consisted of eight networks. A number of dynamic path models were fitted to different sub-networks of these networks: each connected component, each rooted subtree (that is, each gene with all of its descendants), and each interaction separately. For ten models there was at least one significant indirect effect, indicated with rectangles of different colors. Below the thinned forest, the ten models with at least one significant indirect effect are shown. Interactions with significant direct or indirect effects are marked with red arrows. The plus and minus signs on arrows between two genes indicate transcriptional activation and repression, respectively, whereas the plus and minus signs on arrows pointing to survival (d*N*(*t*)) indicate that poor survival is associated with activation and repression of the gene, respectively. For each significant path, the average strength of the direct and indirect effect during the first five years is listed, along with a 95% bootstrap confidence interval.

The diffuse large B-cell lymphoma (DLBCL) data set from [24] contains 7,399 gene expression measurements of 240 patients with DLBCL. Based on a gene list of 1,000 survival genes, 385 genetic interactions were generated. Nine of these were chosen by the selection procedure (Figure 6), which were much higher than expected by chance alone (Table 1). The thinned survival forest consisted of eight networks.

Four dynamic models with at least one significant indirect effect were found (Figure 6). All models were simple, consisting of two genes, and in two cases the direct effect was not significant (models 16 and 17). Both strengthening and counteracting indirect effects were found. The direct effect of *MYC *(0.024) was strengthened by the indirect effect caused by repression of *GAS1 *(0.003), increasing the total effect of *MYC *to 0.027 (model 15). The direct effect of *CCL3 *(0.027), on the other hand, was counteracted by the negative indirect effect through *CCR5 *(-0.016), resulting in a total effect of 0.011 (model 14). The indirect effect of *MYC *and *CCL3 *had strengths of 59% and 13% of the direct effect, respectively.

## Discussion

We have developed a statistical tool based on dynamic path modeling of gene expression data to detect and quantify indirect effects of genes on survival. The use of the additive, rather than multiplicative, hazard model for regression of survival data onto covariates enabled separation of direct and indirect effects in the dynamic path model [20]. By use of permutation tests we demonstrated a high reliability in the selected genetic interactions. Moreover, all possible path models were considered in a systematic way to ensure that all significant effects were detected. Based on three publicly available microarray data sets, we found evidence for significant indirect effects of many transcription factors associated with the survival of cancer patients. Although our findings are clearly data dependent and incomplete, since the identification of effects was based on known transcriptional interactions, they demonstrate how novel information of transcription factor-target interactions and their importance for survival can be obtained with our method. Extending our knowledge of transcriptional interactions may, therefore, increase the number of indirect effects detected, even based on the same expression data.

Confounding represents a persistent danger in studies like ours. We have argued that our method is robust with respect to the most important possible pitfalls. The results are guarded against omitted mediators. Omitted common causes can confound direct and indirect effects, but we have argued that a genetic origin of these is unlikely.

The regulatory networks of many of the transcription factors with indirect effects in our work, such as the PPAR proteins, *E2F1*, *MYC*, and *RUNX3*, are highly complex with numerous interconnected genes and feedback loops [25-29]. Activation of these pathways collectively promotes tumor growth and progression, although expression of the individual members of the pathways is not necessarily associated with survival. The dynamic path models are simple compared to the entire network of the transcription factors, showing that only a few of the interactions are associated with survival in our data. By finding significant indirect effects, we identified key interactions, pointing to the most important pathways. Moreover, the quantitative information of these effects indicates to what extent they counteract or strengthen the direct effect. Note that while the absolute values of the coefficients can be directly compared within each of the data sets, these values are not comparable between data sets, since the data sets are not standardized to a common scale. However, relative values, presented as the ratio between the indirect and direct effect or the indirect and total effect can be compared both within and between studies. The indirect effects contributed significantly to the total effect, and their identification may, therefore, be useful for understanding the role of transcription factors in the development of aggressive tumor phenotypes.

*PPARA*, *PPARD*, and *PPARG *were involved in many of the indirect effects identified in breast cancer. These proteins are members of the nuclear receptor family and are active in the regulation of lipid metabolism, energy balance, inflammation, and atherosclerosis through interactions with numerous genes [25,30]. The participation of these proteins in the most complex dynamic path models was therefore plausible. The indirect effects were mainly mediated through proteins involved in lipid metabolism, such as *ADFP *[31], phospholipid transfer protein [32], and angiopoietin-like protein 4 [33], where the strongest one was the indirect effect of *PPARD *mediated by *ADFP*. A major role of the PPAR proteins in the development of aggressive breast cancers is, therefore, probably to deregulate lipid metabolism through interactions with these proteins. Other transcription factors with indirect effects in breast cancer were *E2F1 *and *STAT5A*, which are essential in the regulation of tumor growth and apoptosis [26,27]. Their indirect effects were mediated through *BBC3 *(*E2F1*), *RAD51 *and *PPARA *(*STAT5A*), suggesting that the interaction of *E2F1 *and *STAT5A *with these proteins contributed significantly to their effect on survival. Of note is the apparent inconsistency between the two breast cancer data sets with respect to the direct effect of *STAT5A*: Repression of *STAT5A *was associated with poor survival in the Dutch data set, whereas activation of the same protein correlated with poor survival in the Uppsala data set. We speculate that this inconsistency could be due to some intrinsic difference in the two populations; for example, patients could be in different stages of the disease for each data set.

*MYC *and *RUNX3*, which are regulators of cellular processes such as proliferation and differentiation [28,29], were among the transcription factors with indirect effects in lymphomas. *MYC *had an indirect effect through the cell cycle inhibitory gene *GAS1*, consistent with previous studies indicating that *GAS1 *repression is important for *MYC*-induced promotion of cell growth [34]. *RUNX3 *showed an indirect effect through the T-cell antigen *CD4*, which is a marker for thymocyte differentiation. *RUNX3 *is required for silencing of *CD4 *[35], and our results suggest that this silencing plays a significant role in *RUNX3*-induced progression of lymphomas.

Many of the transcription factors with indirect effects, including *PPARG*, *E2F1*, *STAT5A*, and *MYC*, have been suggested as targets for cancer therapy [36-40]. The numerous interactions of these transcription factors make the outcome of such targeted therapy difficult to predict. Our work indicates that indirect effects of transcription factors can counteract and thereby diminish the direct effect. This was the case for *PPARG*, *STAT5A*, and *CCL3 *with their indirect effects through *ADFP*, *RAD51*, and *PPARA*, respectively. Such counteracting indirect effects may present severe therapeutic side effects, and caution should therefore be taken before these transcription factors are used as targets. For other transcription factors, such as *E2F1 *and *MYC*, all indirect effects strengthened the direct ones and led to a strong total effect, suggesting that these are more suitable as therapeutic targets. Hence, knowledge of the indirect effects may lead to a better understanding of how targeted therapies involving transcription factors will influence the survival of cancer patients, and, therefore, be helpful for target selection. Moreover, a useful strategy may be to develop compound drugs that target groups of genes simultaneously, to counteract undesired indirect effects. It should also be mentioned that activation of pathways not associated with survival, and therefore not considered here, may induce other side effects that have to be considered in the design of the targeted therapy.

## Conclusion

Dynamic path analysis of large scale gene expression data provides reliable quantification of indirect effects of transcription factors on survival. An improved picture of how transcription factors mediate their effect on survival can therefore be obtained. This may lead to a more precise prediction of the effect of new therapeutics targeting transcription factors and the need to develop complex mixture genetic therapies. Instead of survival, other time-to-event data can be considered in our model, for example, time to relapse of advanced disease. The method can be applied widely and handle other data sets, such as protein phosphorylation data. In such cases, interactions between phosphorylating proteins and their targets will be considered. Gene and protein data can also be included in the same analysis, providing a more comprehensive analysis of pathways.

## Materials and methods

### Path analysis and graphical models

The basic idea of graphical modeling is to represent the relations between variables in a graph with vertices and edges connecting vertices [41]. Vertices represent variables, and there is an edge between two vertices if the corresponding variables are related. The type of relation varies with the specific scientific context and can represent, for example, translational pathways. We focus on DAGs. In a 'directed graph' the edges are directed from one vertex to another. A 'path' is a list of vertices connected along directed edges. A 'directed cycle' is a path of edges beginning and ending in the same vertex, representing a feedback control system. Such regulatory systems are not handled by our method. A directed graph is a DAG if it has no directed cycles.

Each vertex of a DAG represents a random variable *X*_*i*_, where *i *= 1,...,*n *and *n *is the number of vertices. In our application, *i *is a gene, *X*_*i *_is a gene expression measurement, and the DAG represents a regulatory network with several signaling pathways. The edges are interactions between the genes. Informally, there is an edge from *X*_*j *_to *X*_*h *_if *X*_*j *_directly influences *X*_*h*_, that is, if the expression of gene *j *influences the expression of gene *h *in some way. Relationships between variables are assumed to be linear. A 'path model' for (*X*_1_,...,*X*_*p*_) is defined by letting

(1) $$X_{j}=\gamma_{0j}+\int_{h=1}^{j-1}\gamma_{hj}X_{h}+\varepsilon_{j};j=2,3,...,p;$$

where the *ε*_*j *_are independent and identically distributed model error terms with expectation zero and variance *σ*^2 ^(we do not need the assumption that these are normally distributed). The *γ*_*hj *_are called 'path coefficients'. The associated 'path diagram' is a DAG *G *with an edge from *X*_*h *_to *X*_*j *_if and only if *γ*_*hj *_≠ 0.

For a vertex *X*_*j*_, the 'parents' of *X*_*j *_are defined as the set of *X*_*h *_for which there is an edge from *X*_*h *_pointing to *X*_*j*_. Because of the linear structure of equation 1, the path coefficients *γ*_*hj *_can be estimated by recursively regressing each variable onto its parents using ordinary least squares regression.

### The additive hazard regression model

The additive hazard model [19,42] is a model for regression of censored survival data onto (possibly time-dependent) covariates. The model is an alternative to the proportional hazards model [43], which is a multiplicative model. The additive structure of the model is crucial for separating the direct genetic effects from the indirect ones in the dynamic path analysis.

The outcome of interest is, for our purpose, a counting process *N*_*i*_(*t*) indicating whether death has been observed to occur within time *t *for individual *i *(such that *N*_*i*_(*t*) = 1 if death has occurred at or before time *t*; *N*_*i*_(*t*) = 0 otherwise). We use informally d*N*_*i*_(*t*) for the possible change in the counting process in the infinitesimal time interval [*t*, *t *+ d*t*) and denote by F_*t*- _all information (on deaths, censorings, covariates, and so on) until just before time *t*. The intensity process is then defined as:

*λ*_*i*_(*t*) = *Prob*(d*N*_*i*_(*t*) = 1|*F*_*t*-_).

Letting *R*_*i*_(*t*) be the at-risk indicator for individual *i *(*R*(*t*) = 1 if individual *i *is observed at time *t*, *R*(*t*) = 0 otherwise), the intensity process may be written as:

*λ*_*i*_(*t*) = *R*_*i*_(*t*)*α*_*i*_(*t*)

where *α*_*i*_(*t*) is the hazard rate. If *X*_*i*1_,...,*X*_*ip *_are *p *covariates for individual *i*, the additive regression model takes the form:

*α*_*i*_(*t*) = *β*_0_(*t*) + *β*_1_(*t*)*X*_*i*1 _+ ... + *β*_*p*_(*t*)*X*_*ip*_,

where the *β*_*j*_(*t*) are arbitrary regression functions. We may interpret *β*_0_(*j*) as a baseline hazard, while *β*_*j*_(*t*) is the excess risk at *t *per unit increase of *X*_*ij *_for *j *= 1,...,*p*. Estimation is based on the cumulative regression functions $B_{j}(t)=\int_{0}^{t}\beta_{j}(s)ds$. Estimation of the *B*_*j*_(*t*) is easily carried out using a least squares procedure [19,20,42].

### The dynamic path model

A dynamic path diagram [20] is a DAG where the vertex set is partitioned into a 'covariate set' *V*_*c *_= {*X*_1_, *X*_2_,..., *X*_*p*_}, here consisting of all genes in the network, and an outcome variable, which here is d*N*(*t*). We do not allow edges pointing from the outcome to a covariate.

Estimation in the dynamic path model is done by recursive regressions. Within the covariate set *V*_*c*_(*t*) we are back to classical path analysis, and each covariate is regressed onto its parents by least squares. The survival outcome is regressed onto its parents in the graph using the additive hazard regression model. Estimation is carried out repeatedly, each time information changes on covariates (if covariates are time dependent), deaths or censorings.

**Figure 7:**
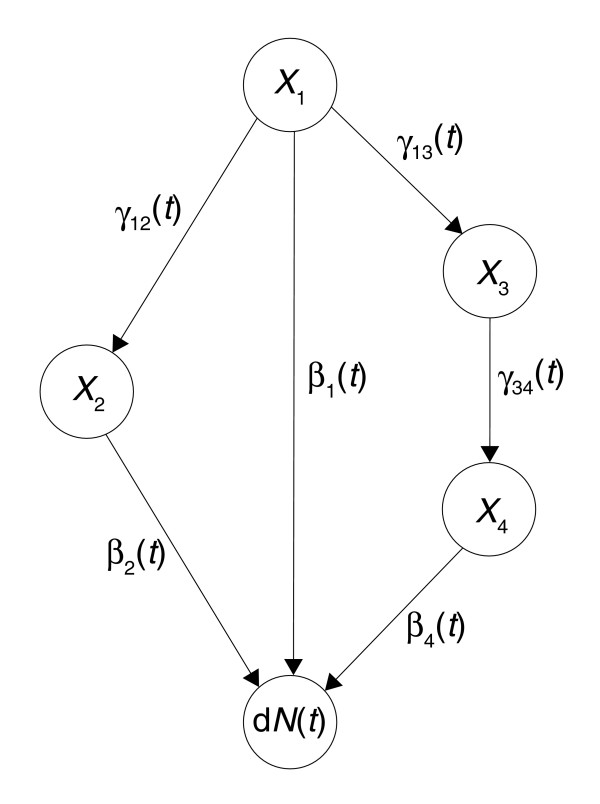
The dynamic path model with direct and indirect effects. This figure shows a dynamic path model with four covariates (genes) *X*_1_,*X*_2_,*X*_3 _and *X*_4 _illustrating how the strength of the direct and indirect effect of *X*_1 _on survival d*N*(*t*) is calculated. Interactions between the covariates are shown as arrows (edges). Each *γ*_*ij*_(*t*) is an ordinary least squares regression coefficient at time *t*, and each *β*_*j*_(*t*) is an additive hazard regression coefficient at time *t*. The strength of the direct effect of *X*_1 _on d*N*(*t*) is given by *β*_1_(*t*). There are two indirect paths from *X*_1 _to d*N*(*t*): one through *X*_2 _and one through *X*_3 _and *X*_4_. The strength of the indirect effect of *X*_1 _on d*N*(*t*) is therefore given by *γ*_12_(*t*)*β*_2_(*t*)+*γ*_13_(*t*)*γ*_34_(*t*)*β*_4_(*t*). The strength of the total effect is the sum of the strength of the direct and indirect effect, that is, *β*_1_(*t*)+*γ*_12_(*t*)*β*_2_(*t*)+*γ*_13_(*t*)*γ*_34_(*t*)*β*_4_(*t*).

Using the dynamic path modeling framework, total effects can be decomposed into direct and indirect effects. A direct effect is an effect that is transmitted through a single edge in the graph to the outcome, while an indirect effect is working through a path of length greater than one (Figure 7). There may be several indirect effects of a gene on the outcome.

More precisely, the direct and indirect effects of a covariate *X*_*h *_on the (survival) outcome d*N*(*t*) are defined as follows. Let there be *r *indirect paths from *X*_*h *_to d*N*(*t*), and denote these by *P*_1_,*P*_2_,...,*P*_*r*_. Let the indirect path *P*_*i *_be of the form:

$$P_{i}=\{(X_{h},X_{i_{2}}),(X_{i_{2}},X_{i_{3}}),...,(X_{i_{w_{i}}},\text{d}N(t))\},$$

where *w*_*i *_is the length of the path. The strength of the indirect effect of *X*_*h *_on d*N*(*t*) is then given by:

(2) $$ind(X_{h}\to dN(t))=\int_{i=1}^{r}\left(\int_{l=1}^{w_{i}-1}\gamma_{i_{l}i_{l+1}}(t)\right)\beta_{i_{w_{i}}}(t),$$

where we let ind(*X*_*h *_→ d*N*(*t*)) = 0 if *r *= 0, that is, if there are no indirect paths, and *i*_1 _= *h*. The definition states that the strength of the indirect effect is the sum over all indirect paths of the products of regression coefficients along each path. The strength of the direct effect dir(*X*_*h *_→ d*N*(*t*)) on d*N*(*t*) is *β*_*h*_(*t*), so the strength of the total effect is given by the sum of *β*_*h*_(*t*) and the expression for ind(*X*_*h *_→ d*N*(*t*)) above (Figure 7). To quantify the strength of the indirect, direct and total effects, we simply plug estimated regression functions ${\hat{\beta }}_{j}(t)$ and ${\hat{\gamma }}_{j}(t)$ into the formulas above. Since each individual time point can be rather noisy, we estimate 'cumulative' direct/indirect effects to improve stability. That is, for ordered event times *T*_1 _<*T*_2 _< ..., the estimated cumulative indirect effect at time *t *is:

(3) $$\bar{Ind}(X_{h}\to N(t))=\int_{T_{k}\leq t}\int_{i=1}^{r}\left(\int_{l=1}^{w_{i}-1}{\hat{\gamma }}_{i_{l}i_{l+1}}(T_{k})\right){\hat{\beta }}_{i_{w_{i}}}(T_{k}),$$

the estimated direct effect is:

(4) $$\bar{Dir}(X_{h}\to N(t))=\int_{T_{k}\leq t}{\hat{\beta }}_{h}(T_{k}),$$

and the total effect is the sum of equations 3 and 4. An example of an indirect and a direct effect estimated in this manner is plotted in Figure 4, which shows the results from one of the models estimated from the Dutch data set. In our data, covariates were time independent; nevertheless, the regression functions are time dependent, since the patient population at risk changes over time.

Confidence intervals of the estimated effects can be calculated using non-parametric bootstrap [44], sampling randomly from the set of all individuals. We have used 1,000 bootstrap replications in our calculations. The percentile method was used for calculating confidence intervals.

### Computation

All computations were carried out using R [45], an open source language and environment for statistical computing and graphics. The additive hazard regression analysis was done using the freely available R package addreg [46]. An R package implementation of our approach is available at [47]. Ordinary linear regression was done using the built-in R function lm. Pathways were generated using Pathway Studio [8].

## Abbreviations

ADFP, adipose differentiation-related protein; DAG, directed acyclic graph; DLBCL, diffuse large B-cell lymphoma; PPAR, peroxisome proliferator-activated receptor.

## Authors' contributions

EF and AF conceived the methodology and the study, together with HL. EF implemented the method, performed data analysis and produced the results. HL contributed biological expertise. All authors wrote and approved the final manuscript.
